# Design and comparative characterization of RecA variants

**DOI:** 10.1038/s41598-021-00589-9

**Published:** 2021-10-26

**Authors:** Elsa del Val, William Nasser, Hafid Abaibou, Sylvie Reverchon

**Affiliations:** 1 UMR5240, Microbiologie, Adaptation et Pathogénie, University of Lyon, Université Claude Bernard Lyon 1, INSA-Lyon, CNRS, 11 Avenue Jean Capelle, 69621 Villeurbanne, France; 2 Molecular Innovation Unit, Centre Christophe Mérieux, bioMérieux, 5 Rue des Berges, 38024 Grenoble Cedex 01, France

**Keywords:** Biochemistry, Microbiology

## Abstract

RecA plays a central role in DNA repair and is a main actor involved in recombination and activation of the SOS response. It is also used in the context of biotechnological applications in recombinase polymerase isothermal amplification (RPA). In this work, we studied the biological properties of seven RecA variants, in particular their recombinogenic activity and their ability to induce the SOS response, to better understand the structure–function relationship of RecA and the effect of combined mutations. We also investigated the biochemical properties of RecA variants that may be useful for the development of biotechnological applications. We showed that *Dickeya dadantii* RecA (DdRecA) had an optimum strand exchange activity at 30 °C and in the presence of a dNTP mixture that inhibited *Escherichia coli* RecA (EcRecA). The differences between the CTD and C-tail of the EcRecA and DdRecA domains could explain the altered behaviour of DdRecA. *D. radiodurans* RecA (DrRecA) was unable to perform recombination and activation of the SOS response in an *E. coli* context, probably due to its inability to interact with *E. coli* recombination accessory proteins and SOS LexA repressor. DrRecA strand exchange activity was totally inhibited in the presence of chloride ions but worked well in acetate buffer. The overproduction of *Pseudomonas aeruginosa* RecA (PaRecA) in an *E. coli* context was responsible for a higher SOS response and defects in cellular growth. PaRecA was less inhibited by the dNTP mixture than EcRecA. Finally, the study of three variants, namely, EcPa, EcRecAV1 and EcRecAV2, that contained a combination of mutations that, taken independently, are described as improving recombination, led us to raise new hypotheses on the structure–function relationship and on the monomer–monomer interactions that perturb the activity of the protein as a whole.

## Introduction

**Figure 1:**
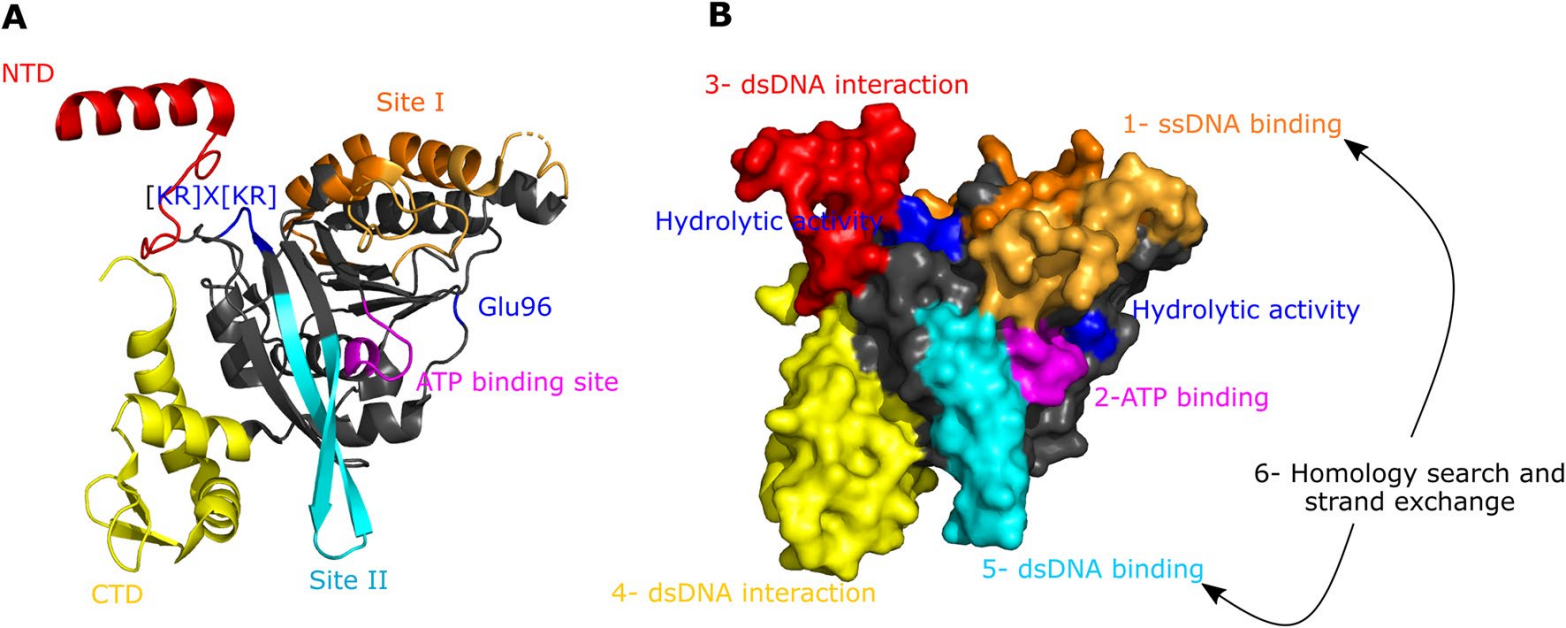
RecA 3D structure and its relationship with the activities involved in recombination. The different domains of the protein are coloured as follows: in red, the N-terminal domain (NTD); in pink, the ATP binding site; in blue, the hydrolytic residue Glu96 and the hydrolytic motif [KR] × [KR]; in orange, site I; in cyan, site II; and in yellow, the dsDNA gateway in the C-terminal domain (CTD). (**A)** RecA structure is shown as a cartoon representation. The different sites are indicated. (**B)** RecA structure is shown as a surface representation. The different steps of recombination are indicated. First, the nucleoprotein filament is formed by binding of single-stranded DNA (ssDNA) to site I (step1). Then, the filament is activated by binding of the cofactor ATP to the ATP binding site (step 2). Next, RecA performs a homology search to find a homologous double-stranded DNA (dsDNA). In this process, dsDNA interacts first with the NTD (step 3) and then with the CTD (step 4), by which it can move to site II, where it binds (step 5). If the bound dsDNA is homologous to the ssDNA, strand exchange is performed; if it is not, the dsDNA is released (step 6). During these steps, the nucleoprotein filament presents hydrolytic activity, carried out by the [KR] × [KR] hydrolysis motif containing Lys248 and Lys250, which cooperate with Glu96 on the other monomer. The figure was generated using the PyMol program^18^ and models provided by Prentiss and Prevost^19^.

Recombinases are responsible for homologous recombination and maintenance of genome integrity, playing a central role in DNA repair mechanisms. In *Escherichia coli*, as in most bacteria, the DNA repair process starts with the formation of a nucleoprotein filament composed of the recombinase RecA associated with the single-stranded DNA (ssDNA) present at a DNA break via site I of the protein and its cofactors ATP via the ATP binding site and Mg^2+^ (Fig. 1). Then, the complex searches for a homologous double-stranded DNA (dsDNA) that can be used as a template for break repair by interacting with and stretching dsDNA. Based on supercoiling, intersegment sampling and clustering of RecA, a genome-wide homology search takes place at a relevant metabolic timescale. In this process, dsDNA interacts first with the N-terminal domain (NTD) and then with the C-terminal domain (CTD), allowing it to move to RecA site II. When a significant homology region is found and stabilized, DNA strand exchange proceeds, forming a heteroduplex complex that is resolved through a combination of DNA synthesis, ligation and resolution. During recombination, the nucleoprotein filament has ATP hydrolytic activity, and this hydrolysis is carried out by the [KR] × [KR] hydrolysis motif containing Lys248 and Lys250, which cooperate with Glu96 on the other monomer. ATPase activity results in a dynamic cycle of binding and dissociation that accelerates the homology search and is essential for promoting extensive DNA strand exchange. Protein flexibility and monomer–monomer interactions are fundamental to the activity of RecA, which functions cooperatively. In addition, many accessory proteins cooperate with RecA, composing a complex network of positive and negative effectors. One of the major accessory proteins is the single-stranded binding protein (SSB) that coats and stabilizes ssDNA and denatures its secondary structure that prevents the formation of nucleoprotein filaments. SSB binds ssDNA with more than 1000 times the affinity of RecA. When RecA filament formation and pairing has been initiated, SSB stimulates DNA strand exchange by binding to the displaced ssDNA^1^. It can also inhibit RecA filament formation by competitive binding to ssDNA^2^.

Furthermore, in *E. coli* system, RecA nucleoprotein filaments promote the SOS response, a coordinated response to major DNA damage. The SOS response is initiated by the accumulation of ssDNA during the replication of DNA-containing lesions. This ssDNA forms a nucleoprotein filament with RecA and ATP. The RecA nucleocomplex then acts as a coprotease to stimulate self-cleavage of the LexA protein as well as other related proteins (phage repressors, UmuD, etc.). LexA is a repressor of a wide variety of SOS genes responsible for a precise and synchronized response that depends on the amount of damage and time since the damage was detected. Self-cleavage of LexA results in derepression of SOS genes. These genes encode proteins that promote DNA repair, damage tolerance and cellular checkpoints to assist cell survival after DNA damage. Among the many SOS genes are the *recA* gene itself, so that via LexA, RecA regulates its own synthesis, and the *sulA* gene. In *E. coli,* SulA acts as a cell cycle checkpoint by inhibiting cell division and thus giving the cell more time to repair DNA damage. Cells survive high DNA damage at the cost of high mutagenesis, which explains the elaborate regulatory control involving the SOS response and RecA activity^3,4^.

Accordingly, RecA is subject to multiple layers of regulation. In fact, RecA did not evolve to have optimized activity but to preserve a balance between different cellular processes and genome stability. Indeed, uncontrolled recombination can interfere with DNA replication and transcription processes^5^. Moreover, bacterial chromosomes frequently carry multiple copies of genes at distinct chromosomal locations, for example, the 7 *rrn* operons in *E. coli,* and uncontrolled recombination can cause genetic damage through the aberrant elimination of genomic segments by recombination between repeated sequences^6^. Thus, there is inherent potential to improve and modify the characteristics of RecA to optimize its activity for in vitro biotechnological applications.

For example, this protein is a major player in the recombinase polymerase isothermal amplification (RPA) used in point-of-care diagnostics^7^. Thus, a structure/function relationship analysis is required to modify its biological and biochemical properties.

In fact, many amino-acid point mutations have been identified to improve the different properties of RecA. Many of them modify the monomer–monomer interface because of the importance of subunit interactions for all RecA activities.

The **L29M** mutation alters the monomer–monomer interface and the flexibility of the whole protein. It is located in the N-terminal region, which is involved in the interaction with dsDNA. The study of chimaeras between RecA proteins of *E. coli* and *Pseudomonas aeruginosa* (Ec-Pa proteins) suggested that this mutation caused a threefold increase in the frequency of recombination exchanges (FRE) in vivo. In vitro*,* it caused an increase in dsDNA affinity, an increase in SSB displacement and a decrease in filament dissociation^8,9^. In the same region and interface of the protein, **R28D** increased the FRE by 38-fold in vivo but was not studied in vitro. However, since the R28A mutation that also increased the FRE (but to an extent that was less than R28D, 27-fold) increased ssDNA binding affinity, we believe that the **R28D** mutation may also have an increased ssDNA binding affinity in vitro^8,10,11^.

Comparison of other Ec-Pa RecA chimaeras highlights other mutations located at the monomer–monomer interface, such as **I102D,** which increases the FRE by twofold, but it has not been studied in vitro. Additionally, the combination of **I159M + S162A + M164V** mutations has the same effects as the mutation I102D, increasing the FRE by 1.5-fold. This combination of mutations also modifies site I of the protein^8^.

Mutations **V79L** and **I102L** have been shown to increase the conjugational capacity in vivo*,* but they displayed a greater persistence on DNA and caused barriers to replication and transcription processes. In vitro*,* they showed a more rapid displacement of SSB. I102L modifies the monomer–monomer interface^12^.

Furthermore, at least one of the **L178I, A179T or L182I** mutations that modify site I of the protein, where ssDNA binds, was supposed to be responsible for the increase in ssDNA affinity of *P. aeruginosa* RecA (PaRecA)^13^. However, this hypothesis has never been investigated in vivo or in vitro*.*

**A289S** was found during directed evolution of enhanced ionizing radiation resistance. It has not been studied in vitro*,* but we hypothesize that it could, similar to D276A or D276N, have an impact on RecA strand exchange activity because these three mutations were found by the same process and have the same in vivo phenotype. It is also thought that this mutation modifies the interaction with the LexA protein. It is located in the CTD of the protein, that is the dsDNA gateway^14,15^.

Finally, suppression of a part of the C-terminus of the EcRecA protein (ΔC17 or ΔC25) has been shown to increase the FRE by fourfold in vivo and to increase dsDNA binding and pairing and to display a more efficient SSB displacement and a more efficient binding to secondary structures in vitro^11,16,17^.

The main objective of this study was to characterize the ability of RecA variants to perform DNA strand exchange reaction required for RPA amplification. Thus, firstly, we highlighted the biological properties of RecA variants, in particular their recombinogenic activity and their ability to induce the SOS response, to better understand the structure–function relationship of RecA and the effect of combined mutations. Then, we highlighted the biochemical properties of RecA variants that may be useful for the development of biotechnological applications and in particular RPA amplification. Indeed, it is fundamentally important to understand the enzymatic mechanisms and to have a panel of different enzymatic properties to design devices that can be used in a wide variety of contexts and applications.

## Results

### RecA variants: selection and design

**Figure 2:**
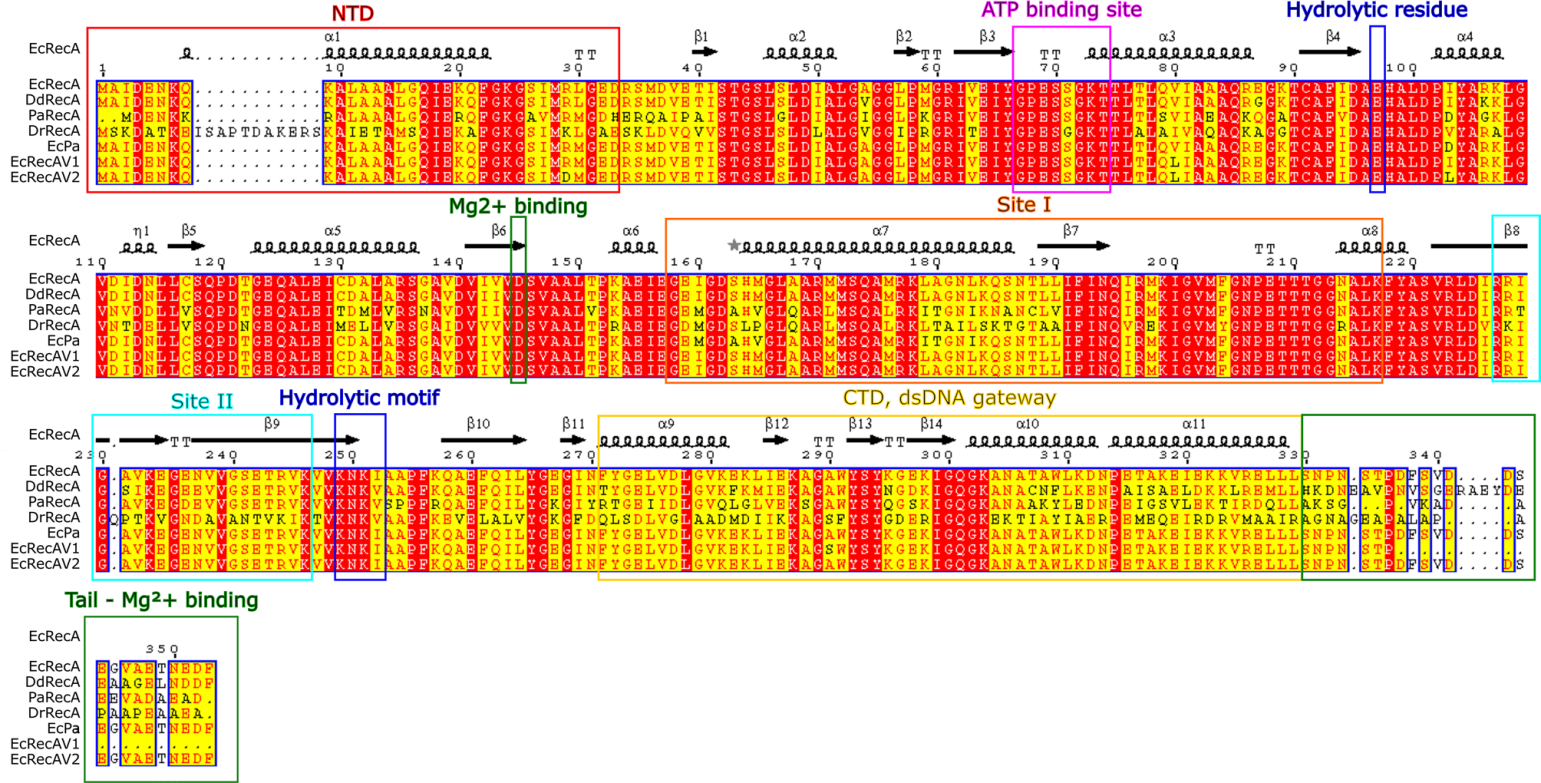
Multialignment of RecA from different bacteria and *E. coli* RecA variants characterized in the study. The sequences aligned are from the following organisms. AML00775: *Escherichia coli* str. K-12 substr. MG1655 RecA; WP_038923633: *Dickeya dadantii* strain 3937 RecA; AAG07005: *Pseudomonas aeruginosa* PAO1 RecA; and AAF11887: *Deinococcus radiodurans* R1 RecA. The three *E. coli* variants EcRecAV1, EcRecAV2 and EcPa were also aligned. The filled red boxes indicate strict identity between residues, and the filled yellow boxes indicate strong homology between residues. No difference is made between black and red characters in this case because this feature is applied for other usages. At the top of the first sequence, the 2D structure of *E. coli* RecA is shown: the squiggles represent α-helices; the arrows, β-strands; the TT letters, strict β-turns; and the star, a residue with multiple conformations revealed by crystallography. The 2D structure is from the PDB entry 4TWZ. Additionally, the different domains of RecA are indicated in boxes: in red, the N-terminal domain (NTD); in pink, the ATP binding site; in blue, the hydrolytic residue and the hydrolytic motif; in orange, site I; in cyan, site II; in yellow, the dsDNA gateway in the CTD; and in green, the C-tail and the Mg^2+^ binding sites. The first methionine is present in the alignments to allow the program to perform well. Thus, the numbering of each amino acid is shifted by one in the figure compared to the text. For example, in EcRecA, L29 is in the 30^th^ position in the figure. The figure was generated using the ENDscript server^27^.

In this work, we selected four RecA proteins originating from different bacteria. EcRecA was chosen as a reference because it is the most studied RecA protein. RecA from *Dickeya dadantii* (DdRecA) was retained because it is a close relative of EcRecA (88% identity, Fig. 2). The major differences are located in the C-terminal domain of these two proteins. However, the two bacteria have different ecological niches and different optimal growth temperatures. While *E. coli* colonizes the gut and grows at 37 °C, *D. dadantii* colonizes plants and grows at 30 °C^20^. Therefore, it is hypothesized that DdRecA may have specific characteristics with regard to EcRecA. PaRecA was included in the study because of its hyperrecombinogenic activity. Indeed, previous works have revealed that in vivo PaRecA has an FRE that is 6.5 times higher than EcRecA^21^. In vitro, PaRecA displays a higher affinity for ssDNA, a more efficient SSB displacement, a higher salt and temperature stability and disassembles at only half the rate of EcRecA^8,9,13^. Finally, RecA from *Deinococcus radiodurans* (DrRecA) was selected because of its distinct biochemical characteristics. *D. radiodurans* is well known for its extreme resistance to ionizing radiation and other DNA damage- and oxidative stress-generating agents. More than 100 genes have been reported to contribute to this resistance in *Deinococcus*. These genes mainly encode proteins involved in DNA repair and oxidative stress defence^22^. DrRecA plays a central role in DNA repair^23^. DrRecA displays 55% sequence identity to EcRecA, with an extension in the NTD and major differences in the CTD, and uses an inverse pathway in which the filament is formed on dsDNA instead of ssDNA. It also tends to create a large number of shorter filaments than EcRecA, while it also nucleates more rapidly but extends the filaments more slowly than EcRecA. Furthermore, it has high DNA binding activity^24–26^.

Additionally, three *E. coli* RecA variants (EcPa, EcRecAV1, and EcRecAV2) were designed. These variants contain a combination of mutations that, taken independently, are described as improving recombination.

The “EcPa” variant is an EcRecA derivative that includes a combination of point mutations corresponding to amino acids naturally found in PaRecA and thought to be responsible for its hyperrecombinogenic activity: L29M, I102D, I159M, S162A, M164V, L178I, A179T and L182I. The “EcRecAV1” and “EcRecAV2” variants include different point mutations that have higher DNA affinity and DNA pairing activity^8,9,11,16,17^ : EcRecAV1 includes the L29M, V79L, and A289S mutations and ΔC17 deletion; EcRecAV2 includes the R28D, L29M, V79L and I102L mutations.

**Figure 3:**
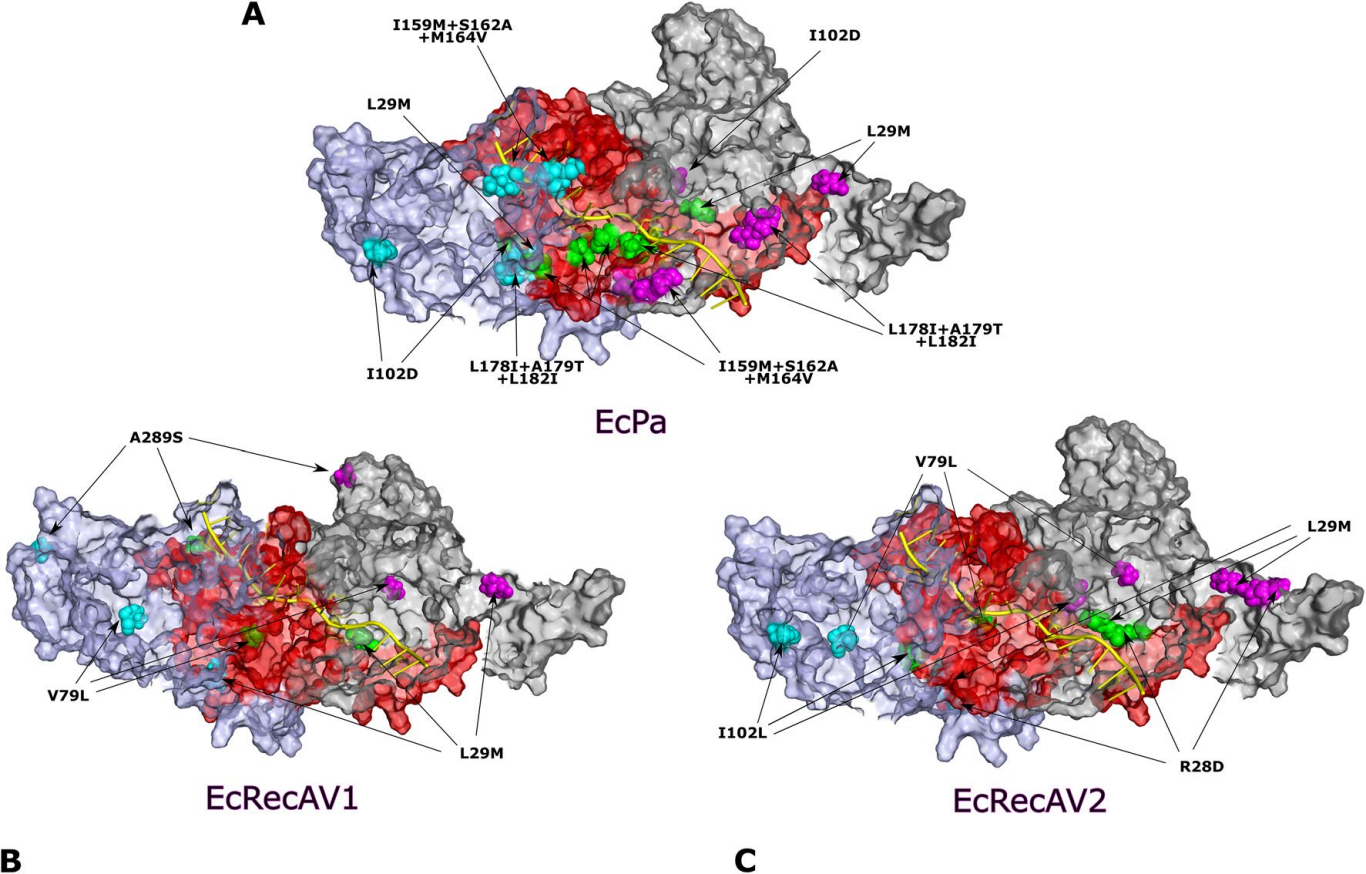
3D structure of RecA variant-ssDNA nucleoprotein filaments. Side view of the 3D structure of 3 monomers of an *E. coli* RecA-ssDNA nucleoprotein filament. Each monomer is coloured violet, red and grey. Amino acids modified in the different variants are represented as spheres and are coloured cyan for the violet monomer, green for the red monomer and pink for the grey monomer. The mutations are indicated by arrows. ssDNA is shown in yellow. (**A**) The EcPa variant includes the L29M, I102D, I159M + S162A + M164V and L178I + A179T + L182I mutations. (**B**) The EcRecAV1 variant includes the L29M, V79L and A289S mutations. This mutant is also truncated by 17 amino acids at the C-tail. As the tail is disordered, it does not appear in the 3D structure. (**C**) The EcRecAV2 variant includes R28D, L29M, V79L and I102L. The figure was generated using the PyMol program^18^ and models provided by Prentiss and Prevost^19^.

Some of these mutated amino acids are close to each other in 3D structure or in direct contact, either within a monomer or at the interface between two monomers, and are susceptible to interaction (Fig. 3). For example, the L29M mutation from monomer 1 is in the vicinity of the I102D or I102L mutation from monomer 2 (mutations present in EcPa and EcRecAV2). Mutations L178I, A179T and L182I from monomer 1 are close to the I159M mutation from monomer 2 (mutations present in EcPa). The L29M mutation from monomer 1 is close to the V79L mutation from monomer 2 (mutations present in EcRecAV1 and EcRecAV2), and finally, mutations R28D and L29M present in EcRecAV2 are consecutive within the same monomer. From the 3D structure, it is difficult to predict whether combinations of these mutations will have additive, synergistic or antagonistic effects on recombination.

The biological and biochemical properties of the seven RecA proteins were studied. In particular, we analysed the effects of RecA production on growth and transcription, their recombinogenic activity and their ability to induce the SOS response*.*

Among the biochemical properties relevant for biotechnological applications, we determined the optimal temperature to be between 30 and 37 °C, the optimal buffer to be between Tris-chloride or Tris–acetate buffer, the impact of the addition of a mixture of the four dNTPs needed in the context of isothermal amplification, as well as ssDNA binding. It was decided to not study the ATPase activity of the different proteins because there is no consistent correlation or coupling between the ATPase activity and the other RecA activities^26^.

### Expression of some RecA variants produces a growth defect

Since the RecA variants engineered in this study have the potential to interact tightly with DNA, we first explored their impact on cell growth. MG1655 nalR *lacZΔP recA*::Cm cells producing any of the seven variant forms of RecA protein expressed under an IPTG-inducible promoter were cultured in LB broth at 37 °C with 0, 0.05 or 1 mM IPTG and 100 µg/ml Ampicillin in microplate. The MG1655 nalR *lacZΔP* RecA^+^ strain transformed with pQE-80L was used as control.

**Figure 4:**
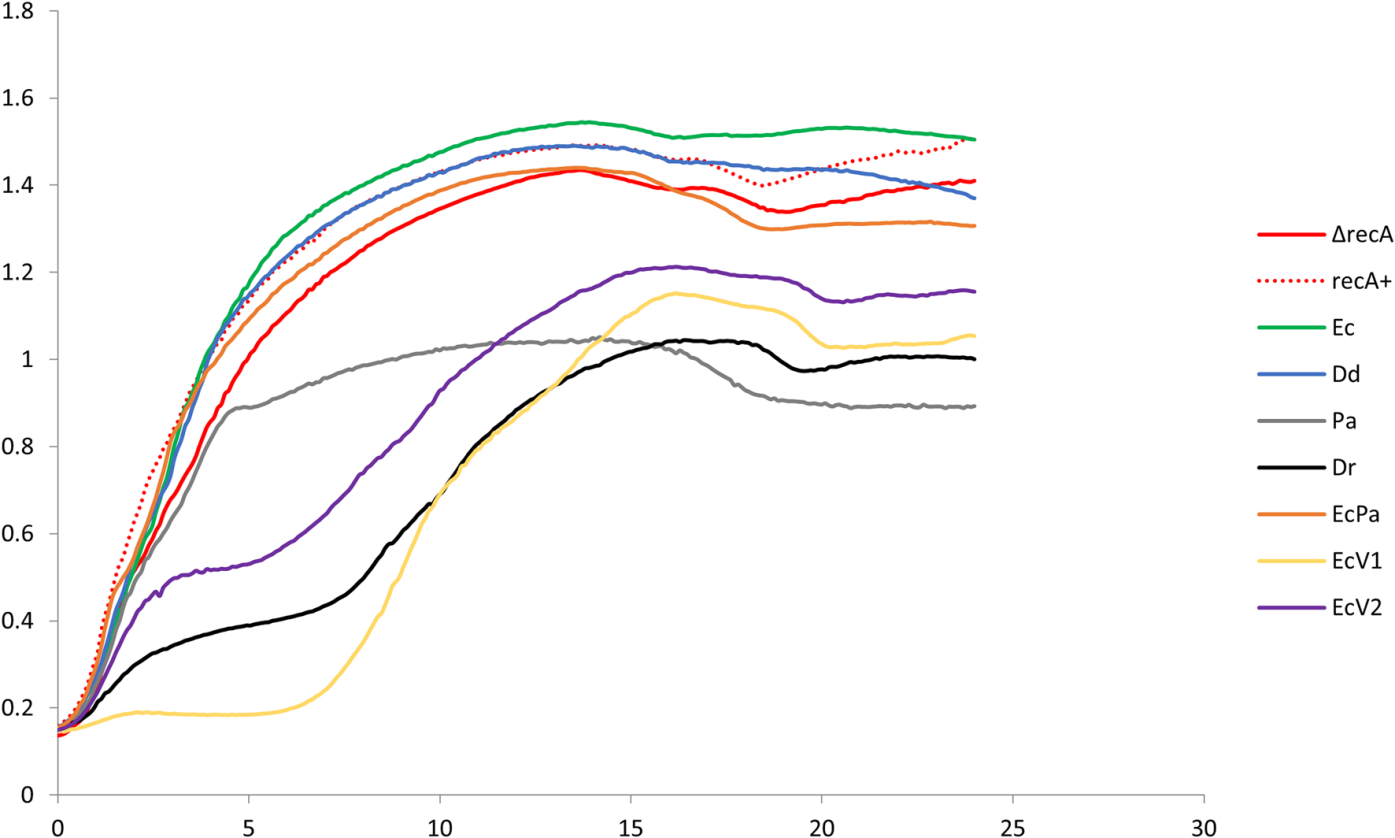
Growth curves of strains producing RecA variants. MG1655 nalR *lacZΔP recA*::Cm cells transformed with pQE-80L plasmid derivatives containing each of the seven *recA* variants induced by 1 mM IPTG were cultured at 37 °C in microplate and the growth was analysed by measuring the OD600 with Tecan Spark for 24 h. Triplicates were performed. MG1655 nalR *lacZΔP* RecA + strain (red dotted curve), ΔrecA strain (red curve), ∆recA strains producing EcRecA (green curve), DdRecA (blue curve), PaRecA (grey curve), DrRecA (black curve), EcPa variant (orange curve), EcRecAV1 variant (yellow curve) or EcRecAV2 variant (violet curve) are shown.

With 1 mM ITPG, relative overproduction of the different RecA proteins ranged from 0.8 to 1.2 compared to EcRecA (Supplementary Fig. 1). Cells producing EcRecA, DdRecA and EcPaRecA showed no growth deficiency, as the growth profile was identical for cells with and without a plasmid expressing RecA (Fig. 4). However, growth was altered in the case of cells expressing PaRecA: the density of cells was lower in the steady state. In the case of cells producing DrRecA, EcRecAV1 and EcRecAV2, growth was even more altered, with a significant lag time before entering the exponential phase, in addition to a lower density of cells at steady state. With 0.05 mM IPTG, toxicity is avoided, except for DrRecA and EcV1RecA for which the toxicity is still present albeit limited (Supplementary Fig. 2). Cellular toxicity could arise due to extreme and potentially deleterious levels of genetic exchange, strong activation of the SOS response that inhibits cell division and indirectly induces toxin-antitoxin production or the creation of barriers to replication and/or transcription by persistently bound RecA filaments^12^.

### Expression of some RecA variants impacts transcription

**Figure 5:**
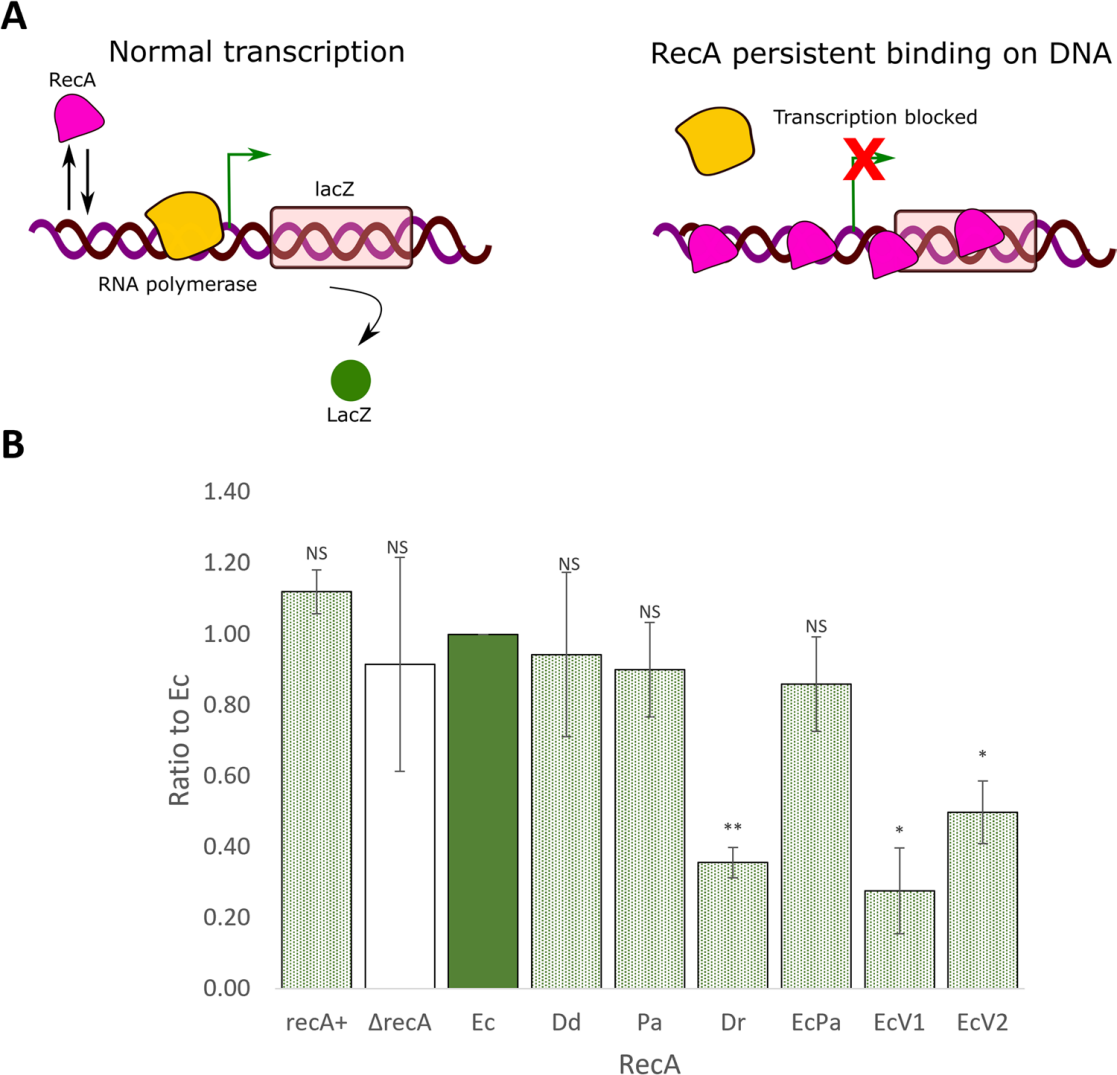
Effect of RecA variants on gene transcription. (**A)** The scheme represents the effect of RecA persistently bound to DNA on transcription: when RecA binds to DNA and then dissociates, the transcription of *lacZ* gene is normal. When RecA is persistently bound to DNA, the transcription is blocked. (**B)** Transcription was assessed by measuring the β-galactosidase activity of MG1655 *recA*::Cm cells transformed with pQE-80L plasmid derivatives containing each of the seven *recA* variants and induced by 0.05 mM IPTG. The ratios of β-galactosidase activity in the presence of the variant versus β-galactosidase activity in the presence of EcRecA, used as a reference, were calculated for 3 different biological experiments. These ratios are represented as a histogram. The strains analysed are RecA + strain (green dot filled bar), ΔrecA strain (unfilled bar), ∆recA strain producing EcRecA (green filled bar, it is the reference but is presented for visual comprehension) and ∆recA strains producing the variants PaRecA, DrRecA, EcPa, EcRecAV1 and EcRecAV2 (green dot filled bars). NS indicated a non-significant p-value (from the one sample t test compared to 1, the EcRecA value), * indicates a significant p-value < 0.05 and ** a significant p-value < 0.005. Error-bars indicate SD.

The capacity of RecA variants to create barriers impacting transcription was evaluated in MG1655 *recA*::Cm (*recA*^*−*^* lacZ*^+^) cells by measuring *lacZ* transcription. This strain was transformed with pQE-80L plasmid derivatives containing each of the seven *recA* variants, induced with 0.05 mM IPTG, a less toxic concentration for growth, and *lacZ* transcription was estimated through a β-galactosidase activity assay. The effect of RecA variants was compared to that of wild-type EcRecA, taken as a reference, by calculating the ratio of β-galactosidase activity in the presence of the variant to β-galactosidase activity in the presence of EcRecA (Fig. 5).

No significant differences were found between EcRecA and the DdRecA, PaRecA or EcPa variant. However, the production of the DrRecA, EcRecAV1 and EcRecAV2 variants induced a significant decrease in gene transcription. This finding suggested that overproduction of these three proteins created barriers that hindered transcription and might contribute to their cellular toxicity.

### RecA variants exhibit different capacities to activate the SOS response

The ability of RecA variants to induce the SOS response was examined by monitoring the expression of the *sulA::gfp* reporter gene. The SOS response was evaluated in two settings: without any induction of the SOS response and after the addition of nalidixic acid, an antibiotic that induces the SOS response.

**Figure 6:**
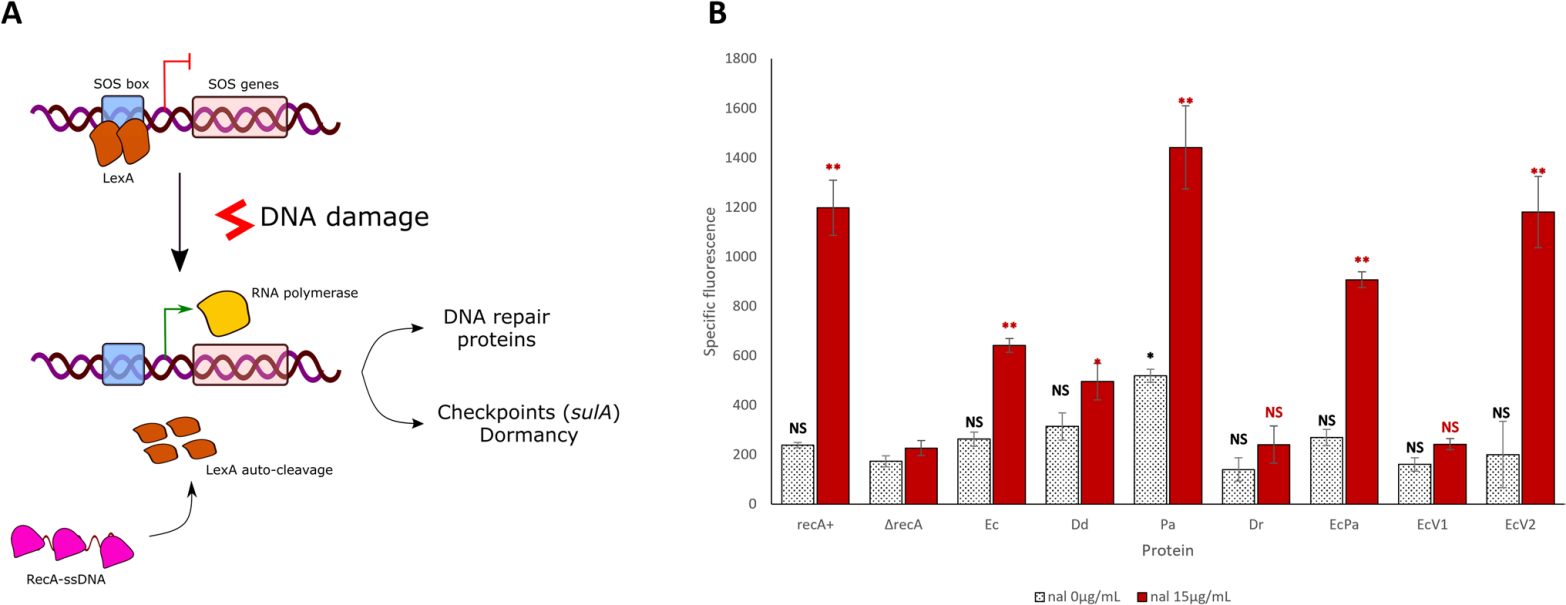
Activation of the SOS response by RecA variants. **(A)** The scheme represents the SOS response in *E. coli*: in the absence of DNA damage, the transcription of SOS genes is blocked by LexA repressor. Under conditions of DNA damage, RecA-ssDNA filaments are generated and catalyse the self-cleavage of LexA. SOS genes, including the *sulA* gene, are then transcribed. (**B)** The SOS response was assessed by measuring the specific fluorescence of strains producing RecA variants under 1 mM IPTG induction and a GFP protein expressed under the *sulA* promoter. Three different biological experiments were performed. The effect of RecA variants in the absence of nalidixic acid is represented in black dot filled bars and the effect of RecA variants in the presence of an inducer of the SOS response, nalidixic acid, at a concentration of 15 µg/mL in red filled bars. The results are presented as histograms. NS indicates a non-significant p-value (from the t test comparing the values of the strains producing RecA variants with those of the strain producing no RecA), * indicates a significant p-value < 0.05 and ** a significant p-value < 0.005. Error-bars indicates SD.

First, none of the proteins produces a constitutive SOS response: in fact, without nalidixic acid, the fluorescence measures were comparable to those without any *recA* expression, with the exception of PaRecA; nevertheless, the fluorescence measured was far lower than in the presence of the inducer antibiotic (Fig. 6).

DdRecA and EcPa variants presented a fluorescence value comparable to that of EcRecA, with and without induction by nalidixic acid. Thus, the SOS response is assumed to be similar for these three proteins and indicates that they are able to interact with LexA. PaRecA resulted in a higher fluorescence value than EcRecA, with and without induction, indicating that this protein induced the SOS more efficiently. The high activation of the SOS response by PaRecA contributed to the inhibition of cellular division and growth deficiency. In the case of the EcRecAV2 variant, fluorescence levels were higher only under nalidixic acid induction. This finding suggested that SOS induction promoted the formation of EcRecAV2 filaments tightly interacting with DNA that were probably unable to disassemble and were consequently responsible for SOS superinduction.

No significant SOS response induction was detected with the DrRecA and EcRecAV1 variants, suggesting that these two proteins were probably unable to interact with *E. coli* LexA.

### Recombinogenic activity of RecA variants

**Figure 7:**
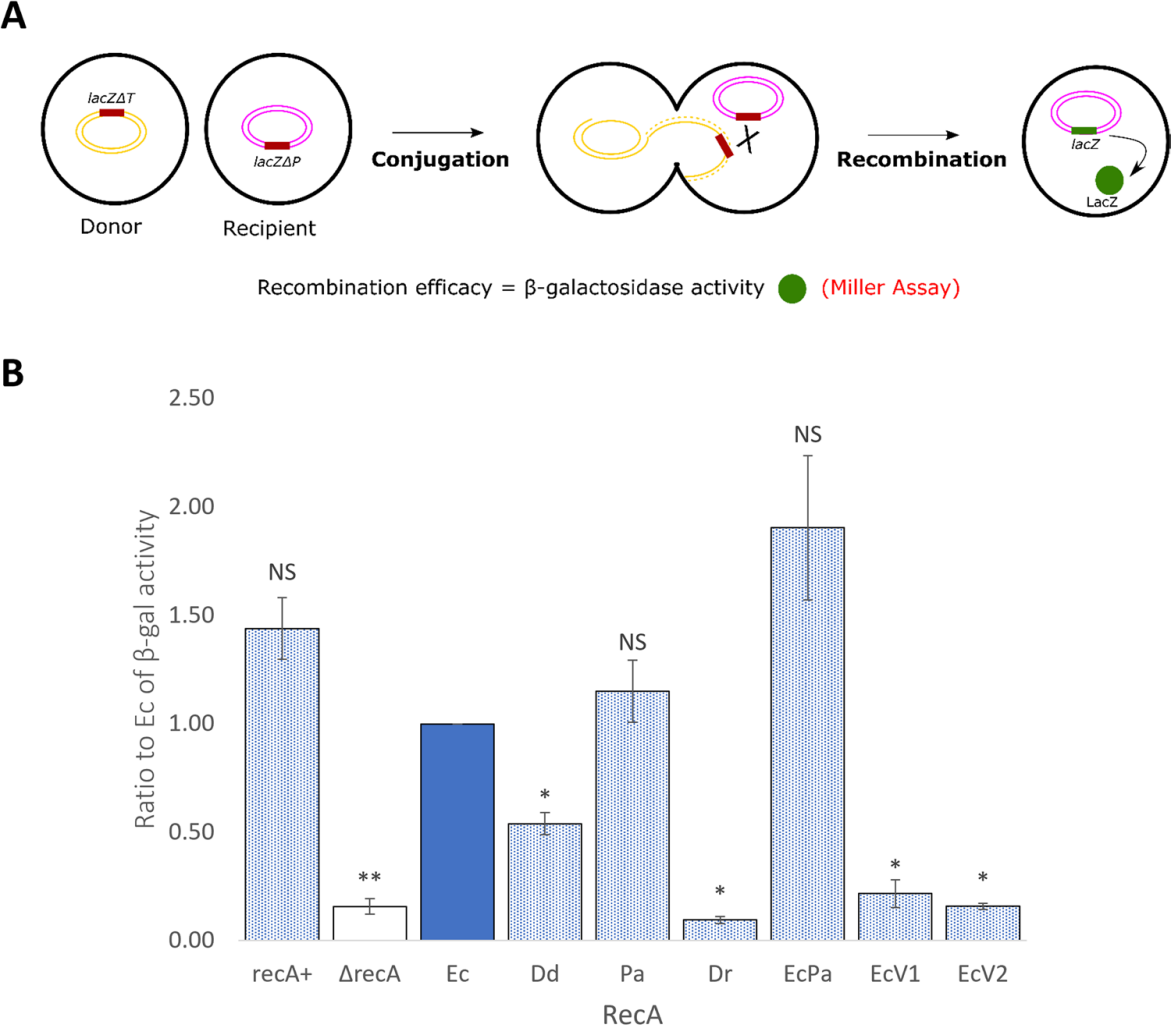
Recombinogenic activity of RecA variants. **(A)** The scheme represents the recombination and reconstitution of *lacZ* gene after conjugation and consequent recombination between the fragments *lacZΔT* of the donor and *lacZΔP* of the recipient. (**B)** Recombination was assessed by measuring the β-galactosidase activity resulting from the restoration of an intact *lacZ* gene after conjugation of MG1655 nalR *lacZΔP recA*::Cm cells harbouring pQE-80L plasmid derivatives containing each of the seven *recA* variants with Hfr3000 *lacZΔT* CmR under protein induction with 0.05 mM IPTG. The β-galactosidase activity was assessed by Miller’s assay. The ratios of β-galactosidase activity in the presence of the variant versus β-galactosidase activity in the presence of EcRecA, used as a reference, were calculated for 3 different biological experiments. These ratios are plotted as a histogram. The strains analysed are RecA + strain (blue dot filled bar), ΔrecA strain (unfilled bar), the ∆recA strain producing EcRecA (blue filled bar, it is the reference but is presented for visual comprehension) and ∆recA strains producing PaRecA, DrRecA, EcPa, EcRecAV1 and EcRecAV2 variants (blue dot filled bars). NS indicates a non-significant p-value (from the one sample t test compared to 1, the EcRecA value), * indicates a significant p-value < 0.05 and ** a significant p-value < 0.005. Error-bars indicates SD.

The recombinogenic activity of RecA variants was estimated by restoration of an intact *lacZ* gene after conjugation between a donor strain Hfr3000 *lacZΔT*::CmR (*lacZ*^*-*^) containing a *lacZ* C-terminal deletion and sharing 2.7 kb homology within *lacZ* with the recipient strain MG1655 nalR *lacZΔP recA*::CmR (*recA*^*−*^* lacZ*^*−*^), which contained a *lacZ* N-terminal deletion. Plasmid derivatives containing each of the seven *recA* variants were introduced in the recipient strain, and conjugation was then performed with the donor strain. Reconstitution of an intact *lacZ* gene was estimated through a β-galactosidase activity assay^28,29^. Recombinogenic activity of the RecA variants was compared to the wild-type EcRecA as a reference by calculating the ratio of the β-galactosidase activity in the presence of the variant to the β-galactosidase activity in the presence of EcRecA (Fig. 7). A conjugation between Hfr3000 *lacZΔT*::CmR and MG1655 nalR *lacZΔP* RecA^+^ was introduced as control.

It is important to note that a lower recombinogenic activity can result from an alteration of *lacZ* transcription caused by some RecA variants, as seen above. However, it was decided to not apply a correction factor to avoid introducing a bias. To exclude the effect on cell growth, traditional recombination assays requiring cell survival were not considered, but transient recombination assays were instead used^30^.

As expected, without any RecA, very low recombinogenic activity was detected. The RecA^+^ control gave similar results to plasmidic EcRecA expressed protein.

The DrRecA and EcRecAV1 and EcRecAV2 variants showed very low recombinogenic activities, which were not significantly different from the negative control (p-values = 0.3192; 0.9656 and 0.4157, respectively, not shown in the figure).

The recombinogenic activity of DdRecA was 2 times lower than that of EcRecA, but the measured activity was significantly different from the negative control (p-value = 0.0015, not shown in the figure).

The recombinogenic activity of PaRecA displayed no significant differences from EcRecA in our transient recombination experimental setup. In the literature, the frequency of recombination exchanges was assayed to be 6.5 times higher for PaRecA than for EcRecA, but the experimental conditions were different. While the monitoring of recombination here was based on Hfr transient conjugation and reconstitution of the *lacZ* gene between donors and recipients, the calculations in the literature were based on the transfer of donor markers after conjugation and selection of transconjugants.

The recombinogenic activity of the EcPa variant tended to be 2 times higher than that of EcRecA, with a p-value of 0.11.

These results will be discussed further below.

### Study of different reaction conditions for the strand exchange activity of RecA variants

The strand exchange activity of each protein was tested in the standard 3-strand exchange assay using M13mp18 substrates in four settings, namely, two buffers, Tris–acetate pH 7.5 and Tris–HCl pH 7.6, classically used in strand exchange reactions (the first is used commonly^11,12,15^ and the second is the commercial buffer developed by New England BioLabs^31^), and two temperatures, 30 and 37 °C, corresponding to the two optimal growth temperatures of the organisms studied.

**Figure 8:**
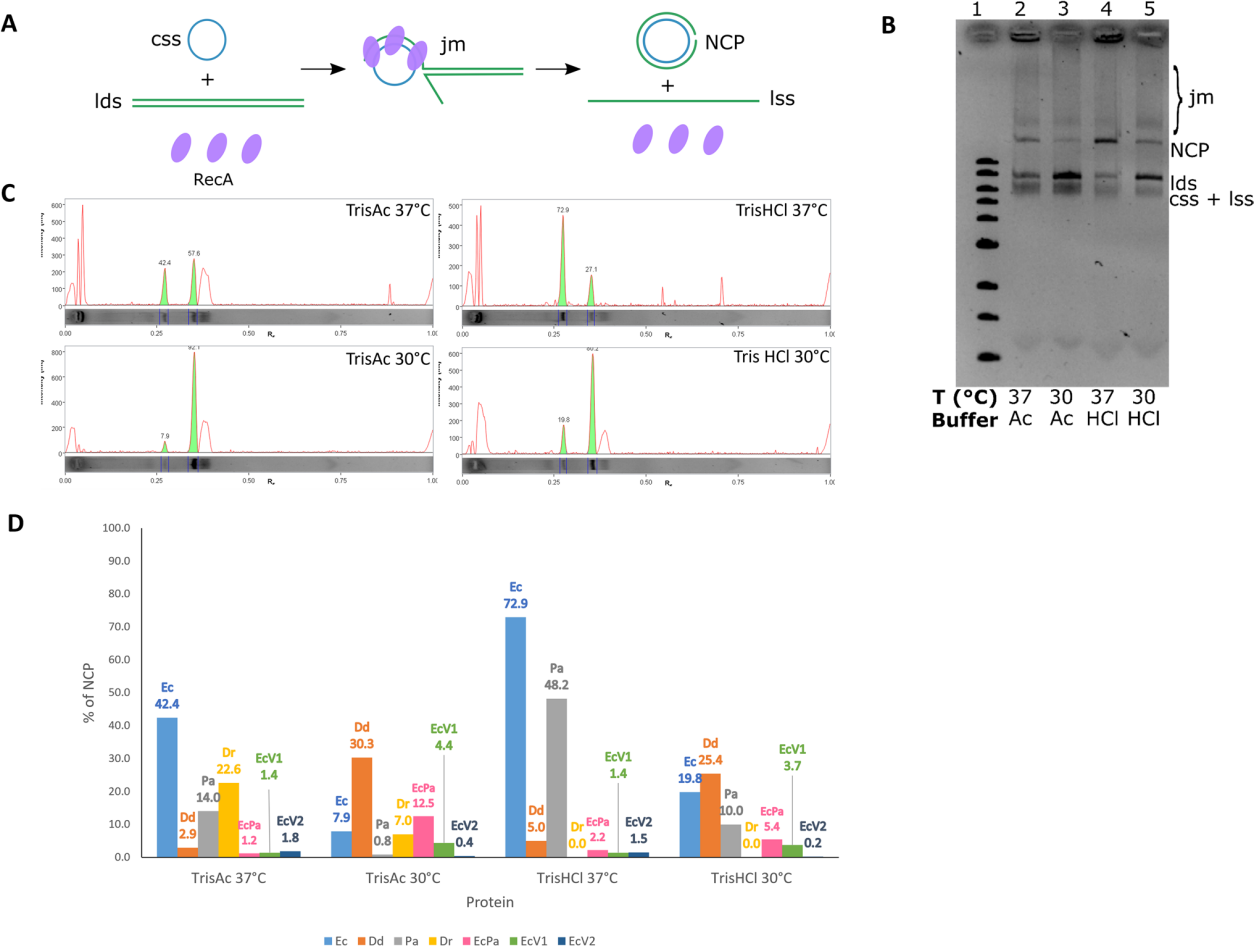
Efficiency of RecA variants in promoting DNA strand exchange. **(A)** Scheme of the three-strand exchange reaction between circular ssDNA (css, in blue) and the linear duplex (lds, in green) substrates and the expected intermediates, called joint molecules (jm), and final products, namely, nicked circular products (NCP) and linear single-stranded DNA (lss), by RecA-mediated DNA strand exchange. (**B)** Example of the DNA exchange reaction obtained with EcRecA in two different buffers and temperatures, indicated above the gel. The first lane corresponds to the 1 kb ladder (full-length gel in supplementary Fig. 1). (**C)** Influence of the buffer composition and temperature on the DNA strand exchange activity of RecA variants. The histogram represents the percentage of fluorescence corresponding to NCP compared to the total fluorescence (NCP plus linear dsDNA; ssDNA and joint molecules were excluded). This result correlates with the efficiency of RecA strand exchange activity. The numbers indicated are the percentages for each condition. The four conditions tested were Tris-Ac buffer pH 7.5 at 37 °C, Tris-Ac buffer pH 7.5 at 30 °C, Tris–HCl buffer pH7.6 at 37 °C and Tris–HCl buffer pH 7.6 at 30 °C for all RecA variants: EcRecA (blue), DdRecA (orange), PaRecA (grey), DrRecA (yellow), EcPa variant (pink), EcRecAV1 variant (green) and EcRecAV2 variant (dark blue).

The efficiency of strand exchange was evaluated semi-quantitatively by calculating the fraction of fluorescence corresponding to the nicked circular product (NCP) compared to the total fluorescence excluding ssDNA and joint molecules (the total fluorescence considered is the sum of the fluorescence of the NCP and the fluorescence of the linear dsDNA) (Fig. 8, Supplementary Fig. 3).

This method allowed us to determine which protein was most efficient in performing strand exchange for each condition, as well as the best buffer and temperature for each protein.

EcRecA was the most efficient variant at 37 °C in both buffers, whereas DdRecA was the most efficient variant at 30 °C.

EcRecA and PaRecA performed best in Tris–HCl buffer, whereas the DdRecA, DrRecA and EcPa performed best in Tris-Ac buffer. EcRecA, PaRecA, DrRecA variants functioned better at 37 °C, whereas DdRecA and the EcPa variants functioned better at 30 °C.

In addition, EcRecAV1 and EcRecAV2 variants were unable to catalyze significant strand exchange independently of the conditions used (< 10%).

The impact of the addition of a mixture of the four dNTPs needed in the context of DNA isothermal amplification for infectious disease diagnostic was further analysed using the same experimental setup and the best conditions established above for the different proteins.

**Figure 9:**
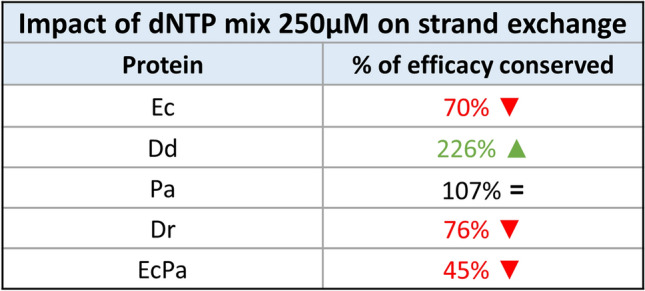
Impact of the addition of the dNTP mixture on the strand exchange reaction catalyzed by RecA variants. The strand exchange efficiency was evaluated for each RecA variant in the presence of a mixture of dNTPs at 250 µM each. Each protein was used under the best conditions determined, except the EcRecAV1 and EcRecAV2 variants, which were not included in this study. The dNTPs were added in the final step of strand exchange, and the efficiency of the reaction was evaluated by calculating the percentage of fluorescence corresponding to NCP compared to the total fluorescence (of NCP plus linear dsDNA; ssDNA and joint molecules were excluded).

The addition of the dNTPs had a negative effect on the activities of EcRecA, DrRecA and EcPa variant, while it had no effect on PaRecA and a positive effect of DdRecA, for which the activity was increased by twofold (Fig. 9). The effects on the EcRecAV1 and EcRecAV2 variants were not evaluated due to the absence of significant strand exchange activity. These results will be discussed further below.

### Study of ssDNA binding to RecA variants

ssDNA binding is the first step performed by RecA and leads to strand exchange and to activation of the SOS response. Altered ssDNA binding can impact all cellular processes involved in DNA repair. Binding of ssDNA should also be evaluated to design effective assays for biotechnological applications.

Therefore, ssDNA binding was assessed by an electrophoretic mobility shift assay. A short 35-nt FAM-labelled ssDNA was incubated with each RecA variant for 10 min under the conditions determined in the previous section. The fraction of bound primer was then determined by fluorescence analysis using electrophoresis on native polyacrylamide gels.

Binding was performed in the presence of ATP or in the presence of non-hydrolysable ATPγS. In the presence of ATP, ATP is hydrolysed, and the filament dissociates, so a low fraction of bound primer was expected. In the presence of ATPγS, the filament is in an active P-state configuration that does not dissociate due to the absence of hydrolysis^32^. Indeed, with ATPγS, RecA filaments have high stability^33^.

**Figure 10:**
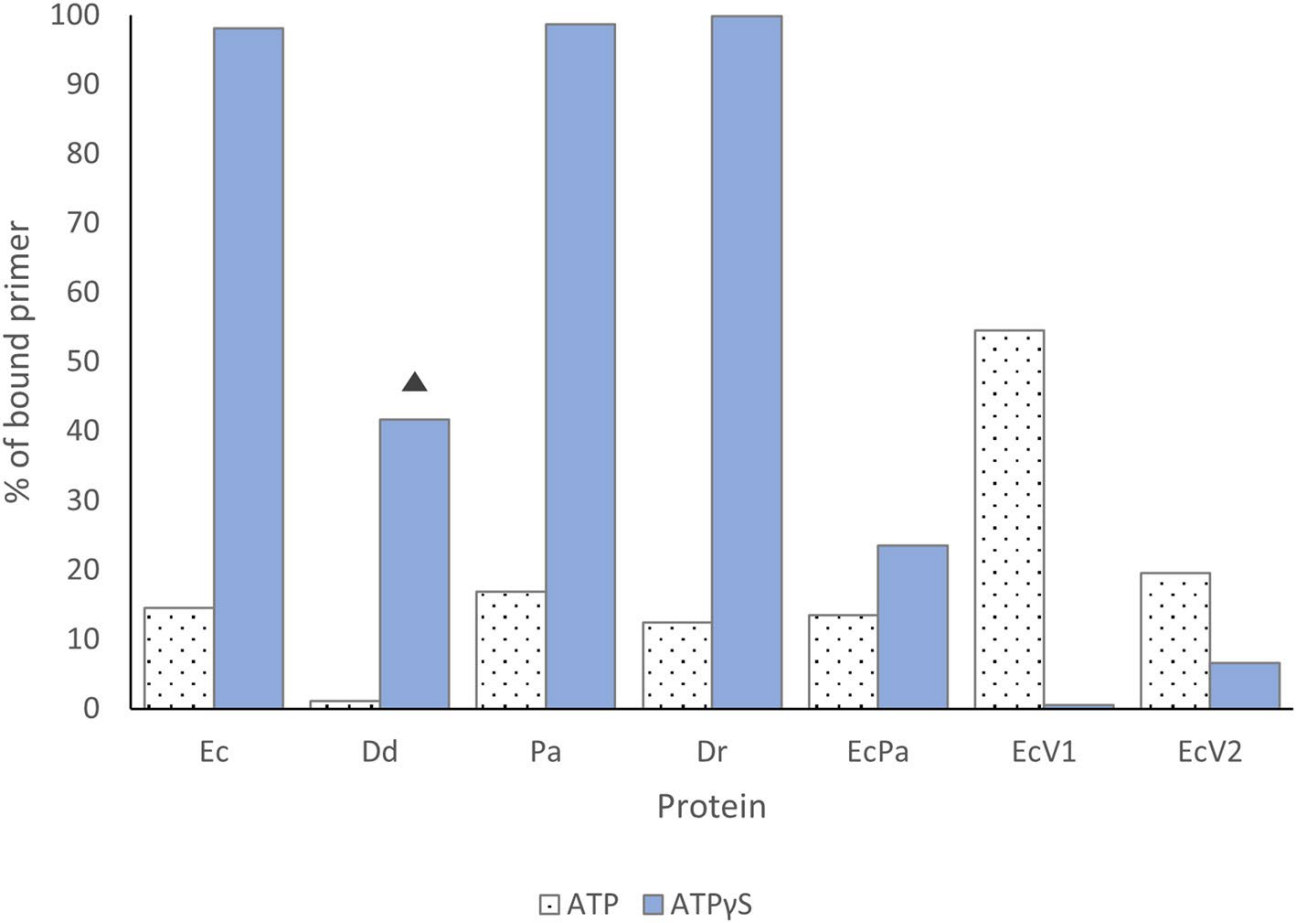
ssDNA binding by RecA variants in the presence of various cofactors. The histogram represents the percentage of fluorescence corresponding to the bound ssDNA compared to the total ssDNA fluorescence. ssDNA binding was assessed in the presence of ATP (white bars with black dots), which is expected to be hydrolysed by RecA and to generate ssDNA-RecA filament dissociation, and in the presence of ATPγS (blue bars), in the presence of which the filament is in a non-dissociating active configuration. The reaction was carried out for 10 min, except for DdRecA in the presence of ATPγS, for which the reaction lasted 20 min because the reaction was observed to be slower (results not shown) (this is indicated by filled triangle in the figure).

Thus, without hydrolysis, binding was optimal for EcRecA, PaRecA and DrRecA (> 90%). Binding was lower but substantial for DdRecA and EcPa variant (> 20%), and it was low for the EcRecAV1 and EcRecAV2 variants (< 10%) (Fig. 10, Supplementary Fig. 4). Note that in the case of DdRecA, binding was assessed after 20 min, rather than 10 min, due to the observed slow binding kinetics (results not shown).

In the presence of ATP, binding was at least fivefold lower than binding in the non-dissociating conformation for EcRecA, DdRecA, PaRecA and DrRecA and twofold lower for the EcPa variant. However, for the EcRecAV1 and EcRecAV2 variants, contrary to expectations, the binding of ssDNA was higher in the presence of ATP than in the presence of ATPγS. These data highlighted an altered cofactor interaction for these two RecA variants.

## Discussion

The RecA protein structure, shared by RecA homologues in virtually all organisms, is highly malleable and tailored by evolution to the requirements for genome maintenance in each particular organism. Indeed, comparisons of RecA proteins from different bacteria highlight subtle variations.

In this work, we compared the biological and biochemical properties of four RecA proteins originating from *E. coli*, *D. dadantii*, *P. aeruginosa* and *D. radiodurans* and three EcRecA variants containing a combination of mutations that, taken independently, are described to improve recombination.

Both in vivo and in vitro results revealed variations in the biological and biochemical behaviours of the RecA variants compared to wild-type EcRecA, which was chosen as a reference.

DdRecA and EcRecA behaved similarly. Their overproduction had no impact on growth and gene transcription. In addition, both activated SOS response. Homologous recombination activity was twofold lower for DdRecA than for EcRecA. As expected from the high sequence similarity, the in vivo activity was similar between DdRecA and EcRecA. However, in vitro, the differences were more pronounced: the activity of DdRecA was optimum at 30 °C, at which temperature the activity was higher than that of EcRecA, which is optimum at 37 °C. This probably reflects an adaptation of RecA proteins from these two enterobacteria to their respective ecological niches during evolution.

Furthermore, the addition of dNTPs at a concentration of 250 µM used for the biotechnological application RPA, which inhibits EcRecA activity, increased DdRecA activity by twofold. Inhibition of wild-type EcRecA by GTP, CTP and TTP is well known and is explained by the competition to occupy the ATP binding site and a decrease in the binding affinity to DNA, affecting filament structure^4,34–38^. As dATP acts as a cofactor for RecA in the same way as ATP, it is hypothesized that the dNTP would interact in a similar way to the corresponding NTP and that adding competitive cofactors to the reaction would explain the inhibition observed for EcRecA strand exchange activity^39^. The positive effect of dNTPs on DdRecA is particularly interesting but could not be correlated with any sequence differences between EcRecA and DdRecA ATP binding sites or hydrolytic residues,. As the CTD and the C-tail of DdRecA are slightly preserved compared to EcRecA, we hypothesized that this domain plays a role in the conformation changes induced by cofactor binding and could explain the modifications of the dNTP effect (Fig. 2). These two findings can be used in the development of isothermal RPA. Indeed, the temperature of 30 °C is closer to room temperature than 37 °C in a large number of countries. On the other hand, a slower interaction with short ssDNA has also been observed, a property that should be considered during the conception of biotechnological applications. As this last property can slow down the homology search between the oligonucleotide and the DNA of the sample, it can also modify the balance with the activity of the polymerase, which could be less obstructed by RecA with this type of kinetics.

Overexpression of PaRecA altered cell growth and induced a higher SOS response than the EcRecA-activated response. PaRecA has been described earlier as lacking constitutive SOS function and having a normal ability to cleave the *E. coli* LexA repressor. Nevertheless, it has been shown that in an *E. coli* genetic context, a higher SOS response is observed: when an operator promoter from the *E. coli lac* gene is used instead of the operator promoter from the *P. aeruginosa recA* gene, the basal SOS response without inducer is 1.8-fold higher than that of *E. coli*, which is explained by the higher protein expression relative to the very weak expression in the *P. aeruginosa* background^8,21^. Thus, our results could be explained by the overproduction of PaRecA from pQE-80L plasmid. In addition, the higher SOS response in the presence of the inducer nalidixic acid confirmed that the SOS response induced by PaRecA is not constitutive. On the other hand, no impact on transcription was found, indicating that PaRecA filaments did not persistently bind to DNA and did not form significant barriers hindering transcription. Homologous recombination activity was close to that of EcRecA. However, the activity was expected to be higher since a ΔFRE of 6.5 has been reported in the literature. This discrepancy can be explained by the difference between the two experimental procedures, highlighting the variability of RecA activity depending on the experimental and cellular conditions. In vitro, the best working conditions of this protein were close to those of EcRecA, except that the activity of PaRecA did not appear to be inhibited by dNTPs. PaRecA, similar to DdRecA, also presents differences in the CTD suggesting that these differences are responsible for the insensitivity to dNTP inhibition. In addition, it is interesting to note that although the optimum growth temperature of *P. aeruginosa* is 30 °C, the RecA activity was higher at 37 °C compared to 30 °C. This may be related to the fact that *P. aeruginosa* is an opportunistic pathogen capable of colonizing the lungs and causes severe infection in individuals with cystic fibrosis. In the lungs, *P. aeruginosa* is confronted with 37 °C temperature and with the host immune response and must deal with oxidative stress that causes DNA damage by various mechanisms. For example, among the reactive oxygen species, the highly reactive hydroxyl radical (•OH) reacts with DNA by addition to double bonds of DNA bases and by abstraction of an H atom from the methyl group of thymine and each of the C-H bonds of 2′-deoxyribose^40^. Optimal activity of RecA is therefore necessary under these conditions to repair DNA. Recently, it has been shown that EcRecA is inactivated under oxidative stress following oxidation of two important methionine residues (Met 35 and Met 164) to methionine sulfoxide. These two residues are not conserved in PaRecA and DrRecA originating from bacteria that are highly resistant to oxidative stress (Fig. 2)^41^.

Overexpression of DrRecA altered cell growth and had a negative impact on gene transcription, indicating that it bound DNA permanently and created barriers to RNA polymerase and probably also to DNA polymerase in the *E. coli* cellular environment. In addition, we observed that DrRecA did not perform homologous recombination in *E. coli,* suggesting that it could not interact with *E. coli* recombination accessory proteins. These behaviours could also be due to the absence of specific proteins interacting with DrRecA in its native cellular environment. In particular, *D. radiodurans,* encodes an unusual SSB that is twice the size of EcSSB and plays a more central role in DNA pairing and strand exchange^42^. In addition, *Deinococcus* encodes several proteins that contribute to extreme radioresistance, including RqkA or PprA, which are absent in *E. coli*. RqkA is a serine threonine/tyrosine kinase that catalyses the phosphorylation of DrRecA at Tyr-77 and Thr-318 and modifies its activity in several ways, including increasing its affinity for dsDNA and its preference for dATP over ATP. It enables DrRecA to use abundant dsDNA substrates for efficient dsDNA break repair^43^. The PprA protein negatively regulates DrRecA functions by inhibiting DNA strand exchange and ATP hydrolysis to protect the *D. radiodurans* genome from hyperrecombination and its associated negative effects^44^.

In the same way, the absence of the SOS response could be due to the absence of an interaction between DrRecA and EcLexA. This can be partly explained by the substitution of arginine, which is involved in the EcReca-LexA interaction, by a lysine in DrRecA at position 243^21,45^. In addition, although *Deinococcus radiodurans* encodes two LexA homologues that possess proteolytic activity that can be stimulated by RecA, these proteins do not participate in the induction of *recA*, indicating the malfunction of the classical SOS response system^46–48^.

Concerning the in vitro activities of DrRecA, the main difference from EcRecA was the buffer specificity. DrRecA was totally inhibited by the Tris–HCl buffer. Its highest activity was found in Tris–acetate buffer, and the activity increased in the presence of glycerol and glutamate potassium (results not shown). In fact, buffer composition affects the biophysical and structural properties of the proteins^52^. The different molecules of the buffer can affect the surface protein and its electrostatic properties, thus having an effect on the protein itself and on the interaction with its different partners. However, because of the extremely diverse properties of proteins, it is difficult to predict a priori the effect of the buffer composition in a theoretical way^52^. Previous studies on the buffer impact on EcRecA have shown that anion present in the buffer influences the binding affinity, which is higher when acetate ions are present instead of chloride ions at the same concentration, suggesting the modification of ionic interactions^33^. Indeed, ion nature and its concentration influence RecA structure, aggregation, unfolding transitions and stability and thus protein–protein interactions^53,54^.

Additionally, similar to PaRecA, DrRecA activity performed better at 37 °C than 30 °C, despite the optimal cell growth temperature of 30 °C. Numerous *Deinococcus* species have been isolated from arid deserts where they are exposed to high temperature and UV radiation^55^, and *D. radiodurans* has been shown to be an extremely resistant bacterium that survives under various environmental stress conditions, including heat stress^56^. The activity of RecA has probably been adapted during evolution to function under these conditions.

The EcPa variant behaved similarly to EcRecA, with unaltered cell growth, gene transcription and SOS response activation. Its homologous recombination activity was twice that of EcRecA. However, in vitro, the activity was not improved compared to EcRecA under the conditions tested. Interestingly, this is an *E. coli* RecA variant with *P. aeruginosa* mutations, both EcRecA and PaRecA proteins being optimal at 37 °C and in Tris–HCl buffer, while the EcPa variant performed better at 30 °C in Tris-Ac buffer. Once again, these results highlight that, the impact of the combination of mutations and the modulation of protein surface properties by different buffers are unpredictable^52^.

As expected, the mutated amino acids present in EcPa led to an increase in homologous recombination activity. This activity is higher than that of PaRecA in this configuration, leading us to believe that the mutated amino acids interact with amino acids only present in EcRecA.

However, it was thought that one of the mutations between L178I, A179T and L182I would modify site I of the ssDNA such that the affinity for ssDNA would be increased^13^. This was not observed; in contrast, the affinity for ssDNA decreased for this variant. Therefore, we hypothesize that these mutations are not responsible for the increased affinity for ssDNA of PaRecA or that they need to interact with other amino acids present in PaRecA but not present in the EcPa variant.

Both the EcRecAV1 and EcRecAV2 variants have a strong impact on cell growth, gene transcription and recombination inhibiting all activities. In vitro, strand exchange was also strongly inhibited. Evaluation of ssDNA binding showed that the in vivo and in vitro observations can be explained by an altered behaviour of these proteins with its cofactor, ATP, in the presence of which DNA binding was strong. This can prevent DNA interactions with the rest of the cellular machinery and be the origin of the inhibition of all cellular processes. Globally, both EcV1RecA and EcV2RecA variants are loss of function mutants. The two proteins have the L29M and V79L mutations that are spatially close in the 3D structure with the L29M mutation from monomer 1 contacting the V79L mutation from monomer 2. Thus, the combination of the two mutations is antagonistic, resulting in an inadequate interaction with the cofactor that causes the absence of homologous recombination activity in vivo and strand exchange activity in vitro*.*

The main difference between EcRecAV1 and EcRecAV2 variants concerned the ability to induce SOS response. EcRecAV1 did not induce the SOS response while EcRecAV2 activated strongly SOS response in the presence of inducer nalidixic acid. The absence of SOS activation for EcRecAV1 could be related to the A289S mutation that has been hypothesized to modify the interaction with LexA^14^. Interestingly, this mutation is also present in DrRecA that does not exhibit the SOS response in the cellular context tested.

On the contrary, the strong SOS response activation by EcRecAV2 variant could be related to the R28D mutation that has been shown to slightly increase the SOS response by 2.2-fold^11^.

In conclusion, we characterized seven RecA variants with different characteristics in vivo and in vitro.

Our study has revealed unexpected effects of combinations of different mutations and that in vivo and in vitro RecA activities do not have direct correlations.

DdRecA was studied for the first time, and three important and distinct features were discovered in vitro: a high activity at 30 °C, the potentiation of strand exchange activity in the presence of a dNTP mixture and the slow binding of ssDNA. These features can be considered in the development of new biotechnological applications, using recombinase polymerase DNA isothermal amplification.

## Materials and methods

### Biochemicals, bacterial strains, enzymes and DNA

The *recA* genes were cloned into the plasmid pQE-80L, with a 6-His tag at the N-terminus, Amp resistance gene and *lacIq* repressor, by Agentide. An empty pQE-80L plasmid was used as a negative control.

The plasmid pZA31-*sulA*-GFP was purchased from Addgene and showed Cm resistance (Addgene plasmid # 78493 ; http://n2t.net/addgene:78493 ; RRID:Addgene_78493)^57^.

The bacterial strains used were Hfr3000 *lacZΔT* CmR (with a *lacZ* gene truncated from its terminal part + 2798 to 3051) and MG1655 *nalR lacZΔP recA*::CmR (with a *lacZ* gene truncated from its initial part − 138 to + 10)^28^. In addition, MG1655 *recA*::CmR was prepared by P1 transduction. For the study of the SOS response, ME12 *∆recA*::kan was used^58^.

For the study of RecA + strains, the strains used are MG1655, MG1655 *nalR lacZΔP* and ME12.

The pQE-80L plasmids were transformed into strain MG1655 *nalR lacZΔP recA*::CmR, strain MG1655 *recA*::CmR and strain ME12 *∆recA*::KanR. In the latter, pZA31-*sulA*-GFP was also transformed (double transformation).

Isopropyl 1-thio-β-D-galactopyranoside (IPTG) was purchased from Euromedex, Ni–NTA resin was purchased from Machery-Nagel (Protino Ni–NTA Agarose), a MonoQ column was purchased from Thermo Fisher Scientific (Pierce Strong Anion Exchange spin column), DNAse I was purchased from Roche, and Bradford reagent was purchased from Bio-Rad (Protein Assay Dye Reagent Concentrate).

M13mp18 ssDNA, M13mp18 RFI and dNTPs (10 mM each) were purchased from New England BioLabs (NEB). M13mp18 RFI was linearized with PstI from NEB. ATPϒS was purchased from Roche. ATP, single-stranded binding protein from *E. coli* (SSB), creatine kinase and phosphocreatine were purchased from Sigma. A 35-nt oligonucleotide labelled with 5’-FAM was synthesized from Eurofins. Double-stranded DNA is a 1-kb preparation from an E. coli gene prepared by PCR amplification and purification with the QIAquick PCR purification kit from Qiagen.

### Effect on cell growth

MG1655 *nalR lacZΔP recA*::CmR transformed strains were used for this experiment. The MG1655 nalR *lacZΔP* RecA + strain transformed with the empty pQE-80L plasmid was used as control. Cells were grown in LB broth at 37 °C with 0, 0.05 or 1 mM IPTG and 100 µg/mL ampicillin in microplate. Cell growth was analysed by measuring the OD600 with a Tecan Spark for 24 h. Each experiment was performed in triplicate.

### Effect on gene transcription

Overnight cultures of MG1655 *recA:*:CmR transformed strains were diluted 50-fold and grown to OD600 = 0.4 in LB broth in the presence of ampicillin at 100 µg/mL. The MG1655 RecA + strain transformed with the empty pQE-80L plasmid was used as control. IPTG was added to a final concentration of 0.05 mM. 0.05 mM was used to avoid the toxic effect on growth. Cells were then grown to OD600 = 1–1.5 and washed in M63 medium. The β-galactosidase activity was measured by Miller’s assay: 100 µL of cells was diluted in 1 mL of buffer Z (60 mM Na_2_HPO_4_, 40 mM NaH_2_PO_4_, 10 mM KCl, 1 mM MgSO4, 0.05 M β-mercaptoethanol, pH = 7) with ONPG to a final concentration of 4 mg/mL. After a 15-min incubation at 37 °C, the reaction was stopped with 500 µL of Na_2_CO_3_ at 1 M and centrifuged for 10 min at 13.2 rpm, and the OD420 was measured. The β-galactosidase activity was calculated in Miller units: 1000 × OD420/(OD600 × volume (0.1 mL) × reaction time (15 min)). Activity was compared with EcRecA activity by calculating the ratio between the two activities. For each strain, three independent biological experiments were performed. For the different RecA variants, the p-value was calculated by performing a one-sample t test compared to 1 (corresponding to EcRecA).

### Effect on the induction of the SOS response

Doubly transformed ME12 *∆recA*::KanR strains were used for this experiment. The ME12 RecA + strain transformed with the empty pQE-80L plasmid was used as control. The SOS response of the different strains was analysed by measuring OD600 and GFP fluorescence (excitation wavelength: 485 nm, emission wavelength: 530 nm) with a Tecan Spark. Cells were grown in LB broth supplemented with 100 µg/mL ampicillin at 37 °C in microplate. After 2 h, IPTG was added to a final concentration of 1 mM. When nalidixic acid was added, it was added after 1 more hour to a final concentration of 15 µg/mL. Measurements were carried out for 22 h. After this time, the final specific fluorescence (defined as the measured fluorescence divided by OD600) was calculated. Three independent biological experiments were performed. The p-value was calculated by performing a t test comparing the variant strain values with the strain without any *recA*.

### Effect on recombination

Overnight cultures of MG1655 *nalR lacZΔP recA::*CmR transformed strains (recipient cells) were diluted 50-fold and grown to OD600 = 0.4 in LB broth supplemented with ampicillin at 100 µg/mL. The MG1655 nalR *lacZΔP* RecA + strain transformed with the empty pQE-80L plasmid was used as control. IPTG was added to a final concentration of 0.05 mM. 0.05 mM was used to avoid the toxic effect on growth. Cells were then cultured to OD600 = 0.8–1. In parallel, an overnight culture of Hfr3000 *lacZΔT CmR* (donor cells) was diluted 50-fold and grown until OD600 = 0.8–1. At this point, the cells were washed in LB, and 1 donor was mixed with 1 recipient, for each recipient cell, in a final volume of 16 mL. This volume was deposited on a nitrocellulose filter (0.22-µm pore size, Merck Millipore). The filters were deposited on a prewarmed LB plate containing 0.05 mM IPTG for 2 h at 37 °C. After this period, cells were resuspended in 2 mL of M63 buffer by vortexing for 1 min. IPTG 0.05 mM, nalidixic acid 40 µg/mL and ampicillin 100 µg/mL were added. Cells were maintained at 37 °C with agitation for 25 h. The β-galactosidase activity was calculated in Miller units as described earlier. A total of 400 µL of cell suspension was used, and the assay was incubated for 24 h to detect low activities. Three independent experiments were performed. For the different RecA variants, the p-value was calculated by performing a one-sample t test compared to 1 (corresponding to EcRecA).

### Protein purification

The recombinant pQE-80L-recA plasmids were transformed into the expression host *E. coli* BL21 (DE3) for overexpression of RecA proteins. The overnight culture was diluted 50-fold in 500 mL of fresh LB medium with ampicillin at 100 µg/mL. The culture was incubated at 37 °C until OD = 0.5–0.6. The culture was then incubated for an additional 2 h at 15 °C until OD = 0.6–0.7. IPTG was added to a final concentration of 1 mM, and the cultures were incubated for an additional 4 h at 15 °C. Cells were harvested by centrifugation, flash frozen and stored at −80 °C. Thawed cells were lysed after resuspending them in 20 mL of lysis buffer (50 mM NaH_2_P0_4_, 300 mM NaCl, 10 mM imidazole, pH 0.8, with protease inhibitor) supplemented with 10 mM MgCl_2_ using a French press. The cell lysate was clarified by centrifugation at 10,000*g* for 30 min at 4 °C. DNAse treatment was performed on the supernatant using DNAse I at a final concentration of 0.5 µg/mL for 10 min at 37 °C. The experiment was continued at 4 °C. The supernatant was loaded onto a column containing 1 mL of equilibrated Ni–NTA resin, and contact was made for 1 h with shaking to allow binding of the proteins to the resin. The lysate was then washed extensively with washing buffer (same as the lysis buffer but with 20 mM imidazole). The bound protein was eluted with elution buffer (same as the lysis buffer but with 250 mM imidazole and 0.1% Triton X-100) in 0.5-mL fractions. After gel analysis, fractions containing a high concentration of RecA were pooled and dialyzed against dialysis buffer (10 mM Tris–HCl, pH 7.3, EDTA 0.1 mM, DTT 1 mM). The sample was then filtered with a 0.45-µm filter and loaded onto an equilibrated MonoQ column. The sample was washed extensively with dialysis buffer, and the bound protein was eluted using a linear gradient of 150–1000 mM NaCl in dialysis buffer. After gel analysis, fractions containing pure RecA were pooled and dialyzed against dialysis buffer and against storage buffer (10 mM Tris–HCl, pH 7.3, 0.1 mM EDTA, 1 mM DTT, 50% glycerol). The protein concentration was checked by the Bradford method using BSA as a standard, and the protein was stored at −80 °C. Protein quality was assessed by 12% SDS-PAGE followed by Coomassie blue staining.

### Buffers

For the in vitro reactions, two different buffers were selected after a literature review and experimental testing (results not shown). The first one corresponded to the NEB RecA reaction buffer (70 mM Tris–HCl, 10 mM MgCl2, 5 mM DTT, pH = 7.6); it is referred to as Tris–HCl buffer. The second, Tris-Ac buffer, was composed of 25 mM Tris acetate, 10 mM Mg acetate, 1 mM DTT, 5% glycerol and 3 mM potassium glutamate, pH 7.5.

### Strand exchange reaction

Twenty microlitres of the reaction mixture containing the selected buffer, 10 µM/nt M13mp18 ssDNA and 10.5 µM RecA (RecA was in excess) was incubated at 30 or 37 °C for 10 min. ATP at 3 mM, an ATP regeneration system (12 mM phosphocreatine and 10 U/mL creatine kinase) and SSB at 0.5 µM were added, and incubation was continued for another 10 min. Finally, linearized M13mp18 RFI l was added at 10 µM/bp. When studied, a dNTP mix was added at this step at 250 µM each. Incubation was continued for 1.5 h. The reaction was stopped by adding 4 µL of 10% SDS and heating at 70 °C for 15 min. Next, 4.8 µL of 6X loading solution was added, and the samples were loaded onto a 0.7% agarose gel and electrophoresed in Tris–acetate buffer at 70 V for 2 h. The products of the reaction were visualized with a Bio-Rad ChemiDoc imager. The bands were quantified with Image Lab software from Bio-Rad.

### ssDNA binding reaction

Twenty microlitres of the reaction mixture containing the selected buffer, 500 nM 35-nt 5’-FAM-labelled oligonucleotide, 10.5 µM RecA (RecA in excess) and 3 mM ATP were incubated at 30 or 37 °C for 10 min. The non-dissociating conformation was analysed by adding 0.3 mM ATPϒS instead of ATP. The reaction was stopped by adding 4 µL of 6X loading solution without SDS, and the samples were loaded onto a 6% polyacrylamide gel and electrophoresed in Tris–acetate buffer at 10 mA for 1 h. The products of the reaction were visualized with a Bio-Rad ChemiDoc phosphorimager. The bands were quantified with Image Lab software from Bio-Rad.

## Supplementary Information

Supplementary Figures.

## Abbreviations

EcRecA: *E. coli* RecA
DdRecA: *D. dadantii* RecA
PaRecA: *P. aeruginosa* RecA
DrRecA: *D. radiodurans* RecA
EcLexA: *E. coli* LexA
ssDNA: Single-stranded DNA
dsDNA: Double-stranded DNA
SSB: Single-stranded binding protein
RPA: Recombinase polymerase amplification
NCP: Nicked circular product
