# Supplementary material for: Design and comparative characterization of RecA variants
Source: Sci Rep. 2021 Oct 26;11:21106. doi: 10.1038/s41598-021-00589-9 (PMC8548320; doi:10.1038/s41598-021-00589-9)
Supplement: Supplementary file 1 — Supplementary Figures. [file 41598_2021_589_MOESM1_ESM.pdf]

Supplementary Information

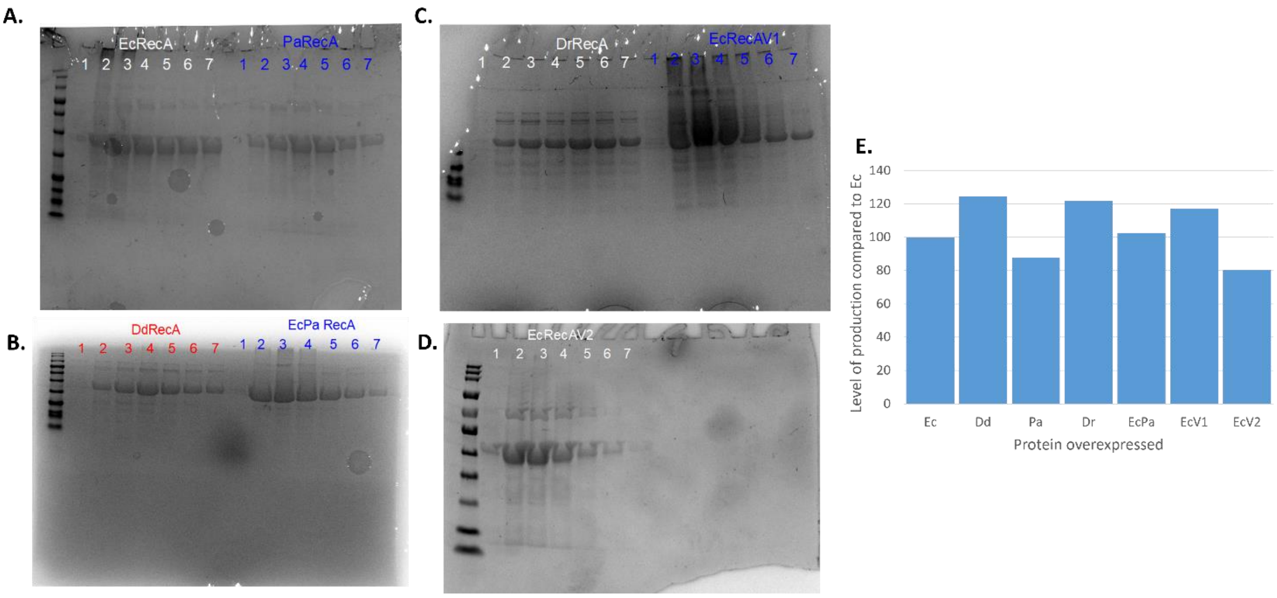

Supplementary figure 1 : Overexpression of the different RecA proteins

Overexpression of the different RecA proteins: A. EcRecA and PaRecA, B. DdRecA and EcPa, C. DrRecA and EcRecAV1, D. EcRecAV2. For each protein, the different protein eluates (1 to 7 indicated in the gel) after NI-NTA Agarose column are analysed in SDS-polyacrylamide gel. The ladder used at the left of each gel is the Blue Prestained Protein Standard, Broad Range of NEB. E. Relative overexpression of the different RecA proteins compared to EcRecA.

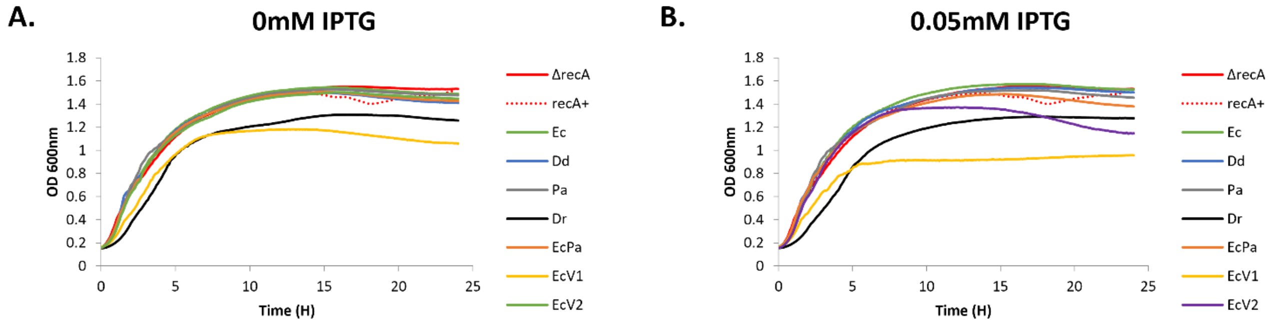

Supplementary figure 2 : Growth curves of strains producing RecA variants

MG1655 nalR *lacZΔP recA*::Cm cells transformed with pQE-80L plasmid derivatives containing each of the seven *recA* variants induced by 0 mM IPTG (figure A) or 0.05 mM IPTG (figure B) were cultured at 37°C in microplate and the growth was analysed by measuring the OD600 with Tecan Spark for 24 h. Triplicates were performed. MG1655 nalR *lacZΔP* RecA+ strain (red dotted curve), ΔrecA strain (red curve), ΔrecA strains producing EcRecA (green curve), DdRecA (blue curve), PaRecA (grey curve), DrRecA (black curve), EcPa variant (orange curve), EcRecAV1 variant (yellow curve) or EcRecAV2 variant (violet curve) are shown.

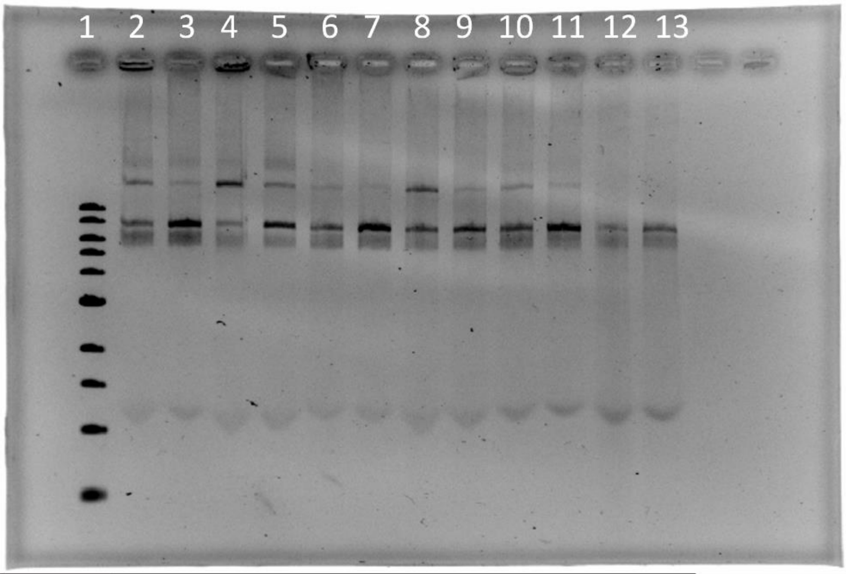

Protein	Ec	Ec	Ec	Ec	Pa	Pa	Pa	Pa	Dr	Dr	Dr	Dr
Buffer	Ac	Ac	HCl	HCl	Ac	Ac	HCl	HCl	Ac	Ac	HCl	HCl
Temperature	37	30	37	30	37	30	37	30	37	30	37	30

Supplementary figure 3 : Efficiency of RecA variants in promoting DNA strand exchange
Example of the DNA exchange reaction obtained with EcRecA (lanes 2 to 5), PaRecA (lanes 6 to 9) and DrRecA (lanes 10 to 13) in two different buffers (acetate and chloride) and temperatures (37°C and 30°C) (for each RecA : 37°C Ac, 30°C Ac, 37°C HCl, 30°C HCl). The first lane corresponds to the 1kb ladder.

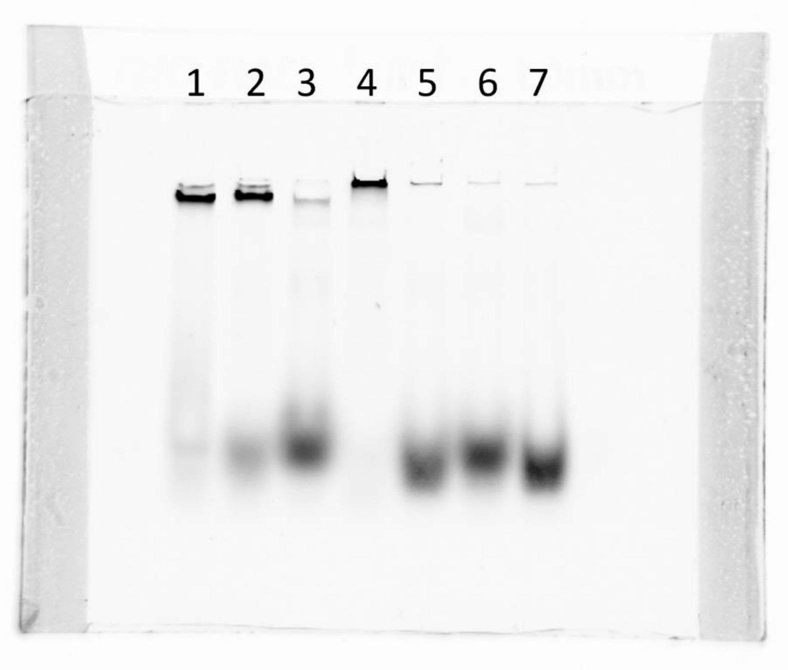

Supplementary figure 4 : Study of RecA-ssDNA binding
ssDNA binding to RecA was assessed in the presence of of ATPγS using a 35-nt 5'-FAM-labelled oligonucleotide. The reaction was carried out for 10 minutes. The results are observed on polyacrylamide gel after fluorescence exposition. The oligonucleotide bound is visible in the top of the gel. The different RecA proteins used are: EcRecA (lane 1), PaRecA (lane 2), DdRecA (lane 3), DrRecA (lane 4), EcPa (lane 5), EcRecAV1 (lane 6) and EcRecAV2 (lane 7).
